# Retinal ganglion cell dysfunction in mice following acute intraocular pressure is exacerbated by P2X7 receptor knockout

**DOI:** 10.1038/s41598-021-83669-0

**Published:** 2021-02-18

**Authors:** Anna Y. M. Wang, Vickie H. Y. Wong, Pei Ying Lee, Bang V. Bui, Stefanie Dudczig, Kirstan A. Vessey, Erica L. Fletcher

**Affiliations:** 1 Department of Anatomy & Neuroscience, University of Melbourne, Melbourne, 3010 Australia; 2 Department of Optometry and Vision Sciences, University of Melbourne, Melbourne, 3010 Australia

**Keywords:** Physiology, Diseases of the nervous system, Ion channels in the nervous system, Neuroscience, Visual system, Retina

## Abstract

There is increasing evidence for the vulnerability of specific retinal ganglion cell (RGC) types in those with glaucoma and in animal models. In addition, the P2X7-receptor (P2X7-R) has been suggested to contribute to RGC death following stimulation and elevated IOP, though its role in RGC dysfunction prior to death has not been examined. Therefore, we examined the effect of an acute, non-ischemic intraocular pressure (IOP) insult (50 mmHg for 30 min) on RGC function in wildtype mice and P2X7-R knockout (P2X7-KO) mice. We examined retinal function using electroretinogram recordings and individual RGC responses using multielectrode arrays, 3 days following acute IOP elevation. Immunohistochemistry was used to examine RGC cell death and P2X7-R expression in several RGC types. Acute intraocular pressure elevation produced pronounced dysfunction in RGCs; whilst other retinal neuronal responses showed lesser changes. Dysfunction at 3 days post-injury was not associated with RGC loss or changes in receptive field size. However, in wildtype animals, OFF-RGCs showed reduced spontaneous and light-elicited activity. In the P2X7-KO, both ON- and OFF-RGC light-elicited responses were reduced. Expression of P2X7-R in wildtype ON-RGC dendrites was higher than in other RGC types. In conclusion, OFF-RGCs were vulnerable to acute IOP elevation and their dysfunction was not rescued by genetic ablation of P2X7-R. Indeed, knockout of P2X7-R also caused ON-RGC dysfunction. These findings aid our understanding of how pressure affects RGC function and suggest treatments targeting the P2X7-R need to be carefully considered.

## Introduction

Glaucoma describes a group of progressive optic neuropathies characterized by the degeneration of retinal ganglion cells (RGCs) and their axons resulting in gradual, irreversible vision loss^1^. Increased intraocular pressure (IOP) is often a major risk factor for glaucoma^2,3^ and reducing IOP is currently the commonest method of disease treatment^1^. Investigating how RGCs degenerate in response to pressure is of critical importance for understanding the detailed mechanisms of glaucoma progression and for identifying novel therapeutic targets.

RGCs are the output neurons of the retina, transmitting signals along their axons to the brain. They are a heterogenous population that can be divided broadly into ON-, OFF- and ON–OFF functional groups based on a response to light increment, decrement, or both^4–6^. Given these functional differences and their structural and molecular underpinnings, understanding how different RGC types are affected by pressure may give greater insights into the degeneration process during glaucoma^7^. An acute, non-ischemic model of IOP elevation induces minimal cell death and is useful for examining individual RGC dysfunction that occurs prior to cell death^8^.

The molecular signalling pathways underlying RGC dysfunction and death during glaucoma remain incompletely understood. Biomechanical and metabolic stressors are thought to trigger a range of cellular pathways that, when overwhelmed, lead to cell death with the purinergic system thought to play a role^9^. Extracellular purines, such as ATP, are known to elicit cellular responses via actions on P2X or P2Y receptors and have been implicated in a range of neurodegenerative diseases^10^.

Increases in ATP concentration have been observed in the eyes of patients and in animal models of glaucoma^11–13^. High ATP levels stimulate the P2X7 receptor (P2X7-R), a non-selective cation channel expressed on RGCs^14–17^. Excessive stimulation of P2X7-R with agonists^18–21^, ischemia^21^ or severe pressure (90 mmHg^21^) lead to cell death in rat tissue and human organotypic cultures. These injuries, however, cause extensive stress to the retina; whereas acute non-ischemic IOP elevation may be better suited to examine the role of the P2X7-R in RGC dysfunction before cell death. Previous studies utilizing acute, non-ischemic IOP elevation have examined the role of the P2X7-R in cytokine release^22^ and RGC morphology in vitro^23^, but effects on RGC function are currently unknown. By using a combination of electroretinogram recordings to measure overall retinal function and multielectrode array recordings to measure individual RGC activity, we can thoroughly assess the role of P2X7-R after IOP injury.

The purpose of this study was to investigate RGC dysfunction following an acute IOP injury and examine whether the P2X7-R plays a role in modulating RGC response. We hypothesized that following acute IOP elevation, dysfunction of specific RGC types might occur and that dysfunction would be minimised in the P2X7-KO mouse. While we found an acute IOP injury caused dysfunction of OFF-RGCs in wildtype mice, ON- and OFF-RGC types, especially, were negatively impacted in the absence of the P2X7-R. Our data suggest that the P2X7-R may play a role in normal ON-RGC function following an acute IOP elevation, indicating that careful consideration is required when exploring this receptor as a target for glaucoma treatments.

## Materials and methods

### Animals

All experiments involving animals were approved by the University of Melbourne animal experimentation ethics committee (Ethics ID number: 1614030) and adhered to the ARVO Statement for the Use of Animals in Ophthalmic and Vision Research and the ARRIVE (Animal Research: Reporting of In Vivo Experiments) guidelines. Animals were housed on a 12/12 h light/dark cycle (on at 8 am, < 50 lx) and fed ad libitum. C57BL/6 wildtype (WT) mice were purchased from the ARC (Animal Resource Centre, WA, Australia) at approximately 8 weeks old and aged at the University of Melbourne. P2X7-KO mice (Pfizer, Groton, CT, USA) were kindly donated by Prof. James Wiley (University of Sydney, NSW, Australia) and bred at the University of Melbourne. Male and female WT and P2X7-KO mice were used at 13 months of age. We have previously shown that the full-length variant 1 of the P2X7-R is no longer expressed in the knock-out mouse, however variants 2/3 and 4 are still present^14^. In addition, the Pfizer P2X7-KO mouse expresses a hybrid P2X7-R that contains a portion of the vector used to disrupt the P2X7 gene, however the remaining variants exhibit lower surface expression and functionality compared to variant 1^24^. Thus, although not a complete knock out of the P2X7-R, the Pfizer P2X7-KO mouse can be used to assess the role of the main P2X7-R variant 1 in RGC function. Adult Thy1-YFP line H (Thy1-YFP-H) mice^25^ were kindly donated by Prof Anthony Hannan (Howard Florey Institute, Parkville, VIC, Australia), bred at the University of Melbourne and used for localization of P2X7-Rs in RGCs. These mice express yellow fluorescent protein in the axon, cell body, and dendrites of ~ 0.2% of RGCs^26^. This sparse labelling allows morphological analysis of the RGC dendritic tree.

### Acute IOP elevation

A total of *n* = 21 WT and *n* = 12 P2X7-KO mice were used. Mice were anesthetised with isoflurane (2% induction, 1% maintenance at 2 ml/min) and placed on a heat pad to maintain core body temperature. Corneal anaesthesia and mydriasis were achieved with a drop of Alcaine (0.5% proxymetacaine hydrochloride, Alcon Laboratories, Frenchs Forest, NSW, Australia) and Mydriacyl (1% tropicamide, Alcon Laboratories). The anterior chamber of one eye was cannulated with a glass micropipette (∼ 50 μm diameter, 1B100–6; WPI, Sarasota, FL) connected via polyethylene tubing (0.97 mm inner diameter; Microtube Extrusions, North Rocks, NSW, Australia) to a pressure transducer (Transpac IV; Abbott Critical Care Systems, Sligo, Ireland) to allow pressure monitoring (Powerlab system, ML110G, and Chart software, ADInstruments, NSW, Australia). This line was also connected to a height-adjustable reservoir filled with Hanks Balanced Salt Solution (Sigma-Aldrich) and to a 5 ml syringe placed in an electronic syringe pump (Harvard Standard Pump 22, Harvard Apparatus, USA) to control fluid infusion (~ 0.001–0.02 ml/min) and maintain pressure at 50 mmHg for 30 min. During IOP elevation, retinae were examined for blanching, as a way of identifying whether this level of IOP affected retinal blood flow. All cannulated eyes were examined and found to be free of any blanching, indicating normal blood flow. This is consistent with a previous study showing that this level and duration of IOP elevation did not decrease retinal blood flow^27^. The contralateral eye was cannulated without any pressure adjustments and acted as the sham control. The eyes were randomised for sham treatment or IOP elevation. Mice were allowed to recover from anaesthesia on a heating pad before being returned to their home cage.

### Electroretinography (ERG)

Three days following cannulation, full-field ERGs were recorded simultaneously from both eyes as previously described^28^. Three days was the chosen time point to maximise RGC dysfunction as recovery has been shown to occur at 7 days post-IOP elevation^27^. The ERG measures the summed responses of photoreceptors, bipolar cells, amacrine cells and RGCs in vivo. The operator was blinded to IOP treatment. In brief, animals were dark-adapted overnight (> 12 h) and prepared for ERG recording with dim red light and infrared night vision scopes to ensure maximal retinal sensitivity. Animals were anesthetised with an intraperitoneal injection of a mixture of ketamine (67 mg/kg) and xylazine (13 mg/kg). Corneal anesthesia and mydriasis were achieved with a drop of 0.5% Alcaine and 1% Mydriacyl, respectively. Animals were lightly secured to a platform and signals were recorded using custom-made chlorided silver active and inactive electrodes (99.99% purity, 0.329 mm ¼ 29 G; A&E Metal Merchants, Sydney, NSW, Australia) that were connected to platinum leads (F-E-30, Grass Technologies, West Warwick, RI). These electrodes were placed on the central cornea (active) and sclera (inactive ring), respectively. These were referenced to a stainless-steel ground electrode (F-E2-60; Grass Technologies) inserted into the tail. Light stimuli across a range of luminous energies (− 5.53 to 2.07 log cd·s/m^−2^) were delivered by an array of 8 white light emitting diodes (LEDs, 8W Luxeon LED, Philips Lumileds Lighting Company, San Jose, CA) and a dim LED (0.1W Luxeon LED, Philips Lumileds Lighting Company) embedded inside a Ganzfeld sphere (Photometric Solutions International, Oakleigh, VIC, Australia). Signals were amplified 1000× and recorded with a band-pass setting of 0.3 to 1000 Hz (− 3 dB) (P511 AC Preamplifier, Grass Technologies) and 4 kHz acquisition (Powerlab 8SP; ADIntruments).

### Analysis of ERG data

The first electronegative component of the ERG waveform (a-wave) in response to the brightest stimuli was modelled using a delayed-Gaussian function^29,30^. The maximal amplitude of the model reflects photoreceptor function (number, outer segment length, or density of non-specific cationic channels). The second and largest component of the ERG waveform (the b-wave) is known to reflect bipolar cell activity. Subtracting the photoreceptor model from raw ERG waveforms more accurately reflect the bipolar cell response. The amplitude of the b-wave was plotted as a function of intensity and modelled with a hyperbolic curve^31^. This model returns a maximum amplitude, which provides an index of ON-bipolar cell integrity.

The scotopic threshold response (STR) is an ERG response measured near absolute light threshold and thought to be generated by inner retinal neurons. The negative STR (nSTR) appears to measure largely amacrine cell function and the positive STR (pSTR) is a good indicator of RGC function in rodents^32^. To improve the signal to noise ratio, peak pSTR amplitude and its implicit time were averaged from parameters isolated from waveforms collected at − 4.9, − 5.01, and − 5.31 log cd·s/m^2^.

We compared the percentage loss of pSTR amplitude, pSTR and nSTR implicit time in WT and P2X7-KO eyes with elevated IOP as these ERG components showed a statistically significant difference in sham and elevated IOP eyes. The difference between IOP and sham eyes was expressed as a percentage of the matching sham eye.

### Multielectrode array recordings

Following ERG recordings, anesthetised mice were sacrificed using cervical dislocation and their eyes were enucleated under dim red light. One eye was taken for perforated multielectrode array (pMEA) recordings and the other eye was processed for immunohistochemistry. The operator was blinded to the IOP treatment. Some WT mice (*n* = 7) did not undergo ERG recordings and pMEA was performed after overnight dark adaptation. Of the eyes taken for pMEA there were *n* = 16 WT and *n* = 12 P2X7-KO successful recordings. For pMEA recording, retinae were dissected in AMES media (A1420; Sigma-Aldrich) supplemented with sodium bicarbonate (1.9 g/l; Chem-Supply, Gilman, Australia) and bubbled with carbogen (95% oxygen/5% carbon dioxide, BOC) to maintain pH at 7.4. The dissection was performed under a combination of dim red light and infrared illumination using night vision goggles attached to a dissecting microscope. Retinae were affixed over a hole in a mixed cellulose-esters membrane (GSWP01300, Millipore) which allowed the photoreceptors to be stimulated after being placed RGC-side down on the pMEA (60pMEA200/30iR-Ti; Multichannel Systems, Reutlingen, Germany). The pMEA used was an array of 64 electrodes, 30 μm in diameter and spaced 200 μm apart. Negative pressure (− 30 to − 35 mBar) was applied using a constant vacuum pump (Multichannel Systems) to draw the RGCs into contact with the recording electrodes. Additionally, a custom titanium harp was used to hold the retina in place on the pMEA and a 13 mm round coverslip was adhered with BluTack (ADHE6000, Complete Office Supplies, Australia) on top of the harp to reduce refractive error from moving AMES medium. The retina was perfused with carbogenated AMES medium at 1.5–1.7 ml/min at 37 °C using a perfusion pump (PPS2; Multichannel Systems), perfusion cannula (PH01; Multichannel Systems) and temperature controller (TC01/02; Multichannel Systems). Retinae were equilibrated under fully dark-adapted conditions for 30 min before recording responses to light stimulation to ensure stable recordings. Using MC-Rack (V 4.6.2, Multichannel Systems), recordings were sampled at 25 kHz and offline analysis was performed on high-pass Butterworth-filtered data (> 200 Hz) to remove full field waveform data and isolate RGC spiking responses.

Spontaneous activity of RGCs was recorded after equilibration and before any light stimulation. To establish the RGC functional responses, visual stimuli were generated using VisionWork for Electrophysiology (Vision Research Graphics, Durham, NH) and provided by an OLED Lucivid display (MicroBrightfieldInc., Williston, VT), focused onto the retina through a 5× Plan-Neofluar microscope objective via a fixed stage, upright Zeiss Axioplan microscope (Zeiss, Oberkochen, Germany). To determine RGC type, square-wave full field stimuli were presented 15 times, with each cycle consisting of 1 s light on and 1 s light off. The intensity was equivalent to 0 and 1125 photoisomerisations/rod/sec during the dark and light phase, respectively.

Receptive fields were assessed using a checkerboard stimulus and the spike triggered average (STA)^33^. Firstly, the retina was adapted to a full field stimulus of 562.5 photoisomerisations/rod/s for 5 s before checkerboard presentation. Then, a 32 × 32 checkerboard was presented and each square (70 µm by 70 µm) was varied in intensity in a binary fashion, either 0 or 1125 photoisomerisations/rod/s, pseudo-randomly using an m-sequence at 12.03 Hz (every ~ 83 ms). Three minutes of responses were collected to ensure that enough data was obtained from stimulated subfields of each RGC to correlate the spikes with the stimulus for the STA to be calculated, revealing the receptive field and allowing calculation of its size.

### Analysis of MEA data

The analysis was performed blinded to genotype and IOP treatment. Spike trains for individual RGC units were determined by spike sorting based on templating and k-means clustering in principal component analysis using Spike2 (v8.07, Cambridge Electronic Design, Cambridge, UK) software. Obvious automatic sorting errors were corrected for each cluster manually. Only cells with < 1% of interspike intervals smaller than 1.5 ms were used for analysis. Spontaneous activity, full-field light responses and STA responses were collected in the same recording, ensuring the cell sorting was performed in the same manner for all stimuli. The time stamps of the RGC action potentials of each sorted unit were exported from Spike2 into MATLAB (R2018b, The MathWorks, Inc., Natick, Massachusetts, United States) for further analysis.

Peristimulus time histograms (PSTHs) in response to full field stimuli and receptive field size analysis from the STAs were assessed using custom MATLAB code. To determine RGC type, full field PSTHs were used and cells showing a clear response to light or dark phase were categorised as ON- or OFF-respectively, or ON–OFF if they responded to both. Otherwise the cell was considered to have no full-field response (NFFR). ON- and OFF RGCs were further classified as transient or sustained cells according to Sagdullaev and McCall^34^ and no difference in the proportion of types were found between-groups (Table S1). As proportions did not differ amongst genotype and IOP-treatment groups, we did not separate ON- and OFF-RGCs into sustained and transient cell types to maintain group sizes and maximise power. The maximum response and spontaneous activity values were determined from the full field PSTHs (binned at 0.02 s) and interpolated using a piecewise cubic hermite interpolating polynomial. The maximum light response of each cell was determined after subtraction of its spontaneous activity measured prior to light stimulus.

Receptive fields were determined through using the binary checkerboard light stimulus. STAs were computed by reverse correlation, which involves aligning the frames of the stimulus sequence that precedes each spike and averaging them^35^. In this case 4 frames preceding each spike were used (1 frame every ~ 83 ms). The 4 resulting STAs from each set of averaged frames were analysed. The receptive fields were detected by applying a Gaussian filter (3 × 3 pixels with 0.5 σ) to the STA, performing edge detection (using the Sobel method and a threshold factor of 1.5). The minimum minor axis length was 1 pixel. The area of the ellipse which appeared to have the highest intensity STA was used as a measure of the size of the field.

### RGC count immunohistochemistry

To determine the effect of acute IOP elevation on RGC numbers 3 days after injury, the eyecups chosen for immunohistochemistry were fixed in 4% paraformaldehyde for 30 min and then cryoprotected in graded sucrose solutions (10%, 20%, 30%) as described previously^14,36,37^. There were *n* = 19 WT and *n* = 11 P2X7-KO eyes successfully collected. The sclera was removed and the retina was rinsed 3 times in 0.1 M phosphate buffer (PB), then incubated with primary antibody, rabbit anti-RBPMS (diluted 1:100, Cat# ab194213; Abcam, Melbourne, Australia) in diluent (0.5% Triton, 0.5% dimethylsulfoxide, 1% normal goat serum, 0.05% sodium azide in PB) for 3 nights at 4 degrees Celsius. Retinae were rinsed 3 times with 0.1 M PB and then incubated in secondary antibody goat anti-rabbit conjugated to AlexaFluor 568 (diluted 1:500, Thermofisher Scientific, VIC, Australia) overnight. Following this, retinae were rinsed 3 times with 0.1 M PB before flat mounting on a glass slide, RGC side up, with Dako mounting medium (Dako, North America, S3023). Retinae were imaged with a Zeiss LSM880 confocal microscope (Carl Zeiss AG, Oberkochen, Germany) using a 20× objective. Images were taken at a single z-position through the ganglion cell layer that captured RGC nuclei. At least 4 images from central and peripheral eccentricities were taken for each retina. RGC nuclei were manually counted in Fiji/ImageJ in a blinded manner (v1.52i, NIH, Bethesda, MA, USA) using the Cell Counter plugin (https://imagej.nih.gov/ij/plugins/cell-counter.html). Mean central and peripheral counts were obtained and then averages were calculated for each group (WT sham, WT IOP, P2X7-KO, P2X7-KO IOP).

### P2X7-R immunohistochemistry

To localise P2X7-Rs on RGCs, 3-month-old Thy1-YFP-H mice were used. Immunohistochemistry was performed as above using rabbit anti-P2X7 receptor (1:500; Cat. No# APR-008; Alomone, Jerusalem, Israel) as the primary antibody. The secondary antibodies were run overnight: goat anti-rabbit conjugated to AlexaFluor 647 (diluted 1:500, Thermofisher Scientific, VIC, Australia) and goat anti-green fluorescent protein (1:400, Cat# 600-141-215, Rockland Immunochemicals, Gilbertsville, PA) to enhance the Thy1-YFP-H labelling^37^. A Zeiss Pascal confocal microscope was used to image immunolabelled tissue samples (Carl Zeiss AG, Oberkochen, Germany). RGCs were first classified using a X40 oil objective, and primarily A-type ganglion cells were selected (characterised by their large soma and dendritic field size^38^). RGCs were identified as ON cells if they stratified within 0–40% of the IPL depth and as OFF-cells if they stratified within 60–100%. For analysis of P2X7-R expression in Thy-1 positive ganglion cells, confocal Z-stacks, 20 to 40 optical sections deep (1.4 µm optical section thickness) were collected using the X63oil objective at high resolution (2048 px × 2048 px) with scan speed 6 and averaging of 4. Due to the time required for imaging (2 h per cell), only the cell soma and usually one quarter of the dendrite field were imaged for analysis. Three dimensional images were analysed using the Colocalization module of IMARIS software (×64, v.7.6.5 Bitplane AG, Zürich, Switzerland)^36,39^. IMARIS colocalization analyses the entire two-channel confocal stack by measuring the intensity of each fluorescent label in each voxel (volume pixel). A voxel is defined as having colocalization when the intensities of both labels are above their respective thresholds. P2X7-R puncta colocalised with Thy-1 positive RGCs (co-labelled voxels) were defined as individual puncta using the Spot module of IMARIS and counted based on stratification of the Thy-1 positive RGC, within each layer of the IPL. Following identification of the RGC type, dendritic length and area within each layer of the IPL were measured using MetaMorph software (Molecular Devices, Sunnyvale, CA). Data presented as P2X7-R puncta per 100 µm of RGC dendrite.

### pMEA immunohistochemistry

To observe RGC and retinal morphology after pMEA, a WT retina was gently removed from the pMEA following recording. Whilst still affixed to the filter paper the retina was fixed and cryoprotected as described above. Following this, the retina was incubated with primary antibody rabbit anti-RBPMS (diluted 1:100, Cat# ab194213, Abcam, Melbourne, Australia) and IB4 conjugated to AlexaFluor 647 (1:300, Cat# I32450, Thermofisher Scientific, VIC, Australia) for 4 nights at 4 degrees Celsius. The retina was washed then incubated overnight in secondary antibody goat anti-rabbit conjugated to AlexaFluor 594 (diluted 1:500, Cat# ab150080, Abcam). A tiled image was taken with a Zeiss LSM880 confocal microscope (Carl Zeiss AG, Oberkochen, Germany) using a 10X objective. A Z-stack was taken through the inner retina (7 slices, 6 µm optical section thickness) and processed with the Maximum Intensity Projection module using Zen Black Software (Zen 3.0 SR (black), 16.0.1.306, Carl Zeiss).

### Statistical analysis

All data are shown as mean ± SEM. Statistical analysis was undertaken in GraphPad Prism (v6.01, GraphPad Software, San Diego, CA). ERG data and percentage loss data between sham and IOP eyes were compared using paired t-tests. Statistical analysis of MEA data between sham and treatment eyes were performed using the Mann–Whitney non-parametric test as there was a non-Gaussian distribution of the RGC responses likely due to multiple RGC types grouped in ON-, OFF- and ON–OFF RGC categories. One-way analysis of variances, followed by Tukey’s multiple comparisons test, was used to assess changes in P2X7-R expression in RGCs. Two-way analysis of variances were performed to assess proportions of RGC types and RGC counts in genotype or treatment, followed by Tukey’s multiple comparisons test.

## Results

### RGC electroretinogram responses are reduced following IOP injury

**Figure 1:**
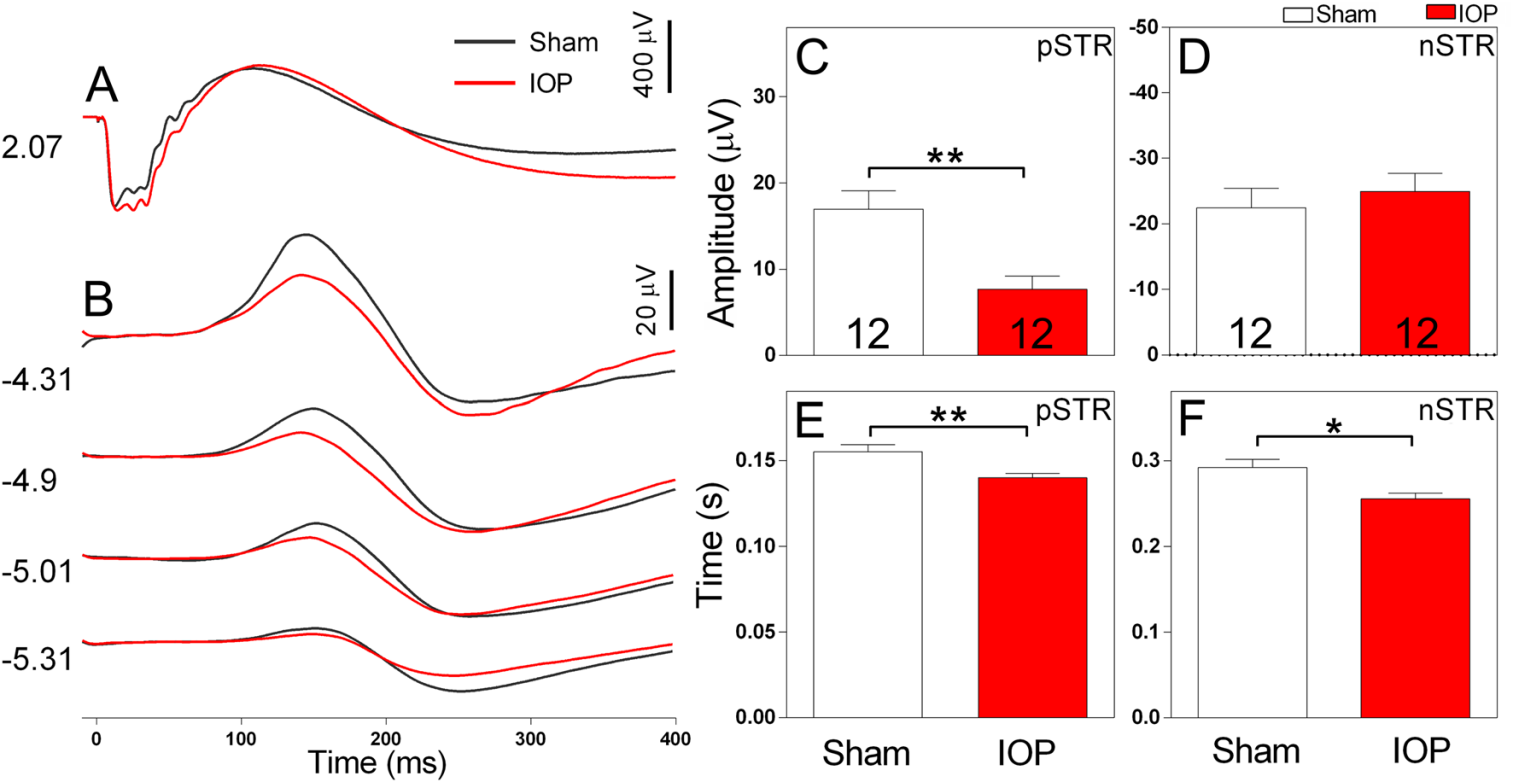
Electroretinogram recordings of WT mice show a prominent reduction the pSTR amplitude (a measure of RGC activity) following acute IOP elevation. Representative waveforms of retinal responses measured at bright ((**A**), 2.07 log cd·s/m^2^) and dim ((**B**), − 5.31 to − 4.31 log cd·s/m^2^) luminous energies. Average amplitude of the pSTR (**C**) and nSTR (**D**) following sham (white bars) or elevated IOP (red bars). Average implicit time to the peak amplitude of the pSTR (**E**) or nSTR (**F**) following sham or elevated IOP treatment. The amplitude and implicit time of the pSTR was significantly reduced in eyes subject to elevated IOP. Numbers indicate the number of animals. *p < 0.05, **p < 0.01; paired t-test (sham vs IOP, within animal).

To examine the extent of retinal dysfunction in our WT mouse model of acute IOP elevation, we used dark-adapted full field flash electroretinogram (ERG). For each mouse, one eye was randomly assigned to receive IOP injury (50 mmHg for 30 min) and the remaining eye was given a sham treatment (cannulation, without IOP elevation). Three days following the IOP injury, we performed ERG recordings (Fig. 1). By isolating components of the ERG waveform (Fig. 1A), the function of cohorts of retinal neurons including photoreceptors, ON bipolar cells, amacrine cells and RGCs (Fig. 1B) were evaluated^40^. While the function of most retinal neuron classes showed only slight differences to sham (Fig. 1A, Table 1), pSTR amplitude was prominently reduced in eyes with raised IOP compared to sham treatment (Fig. 1B). The average amplitude of the pSTR in WT mice was significantly reduced 3 days following raised IOP compared to sham (7.65 ± 1.53 µV IOP vs 16.94 ± 2.13 µV sham, p = 0.005, *n* = 12 mice, Fig. 1C). In contrast, the average amplitude of the nSTR was similar in sham treated and eyes with raised IOP (Fig. 1D). The implicit time to peak amplitude was also reduced for both the pSTR (0.140 ± 0.002 s IOP vs 0.155 ± 0.004 s sham, p = 0.004, *n* = 12 mice, Fig. 1E) and nSTR (0.256 ± 0.006 s IOP vs 0.292 ± 0.009 s sham, p = 0.021, *n* = 12 mice, Fig. 1F).

**Table 1:** ERG measures of a and b-wave amplitude and sensitivity in WT and P2X7R-KO animals.

	WT		P2X7-KO	
	Sham	IOP	Sham	IOP
**A-wave**				
Amplitude (µV)	− 519.36 + 29.70	− 546.70 ± 25.79	− 539.55 ± 42.38	− 490.49 ± 25.96
Sensitivity (log cd.s/m^2^)	3.03 ± 0.05	2.89 ± 0.04^†^	3.08 ± 0.02	2.94 ± 0.04**
**B-wave**				
Max. amplitude (µV)	692.19 ± 37.49	636.10 ± 44.71	803.67 ± 72.81	689.82 ± 46.70*
Sensitivity (log cd.s/m^2^)	− 2.39 ± 0.08	− 2.35 ± 0.08	− 2.55 ± 0.04	− 2.48 ± 0.05

^†^p = 0.025, **p = 0.0128, *p = 0.0297 compared to sham of the same genotype.

To ensure the ERG results from sham eyes were representative of normal retinal function, sham ERG measures were compared to those from age-matched naïve WT mice that had not undergone any prior procedures. A- and b-wave amplitude and sensitivity, and pSTR and nSTR amplitude and implicit time were determined to be similar between the two groups (unpaired two-tailed t-test; Table S2).

### OFF-RGC responses are reduced following acute IOP injury

**Figure 2:**
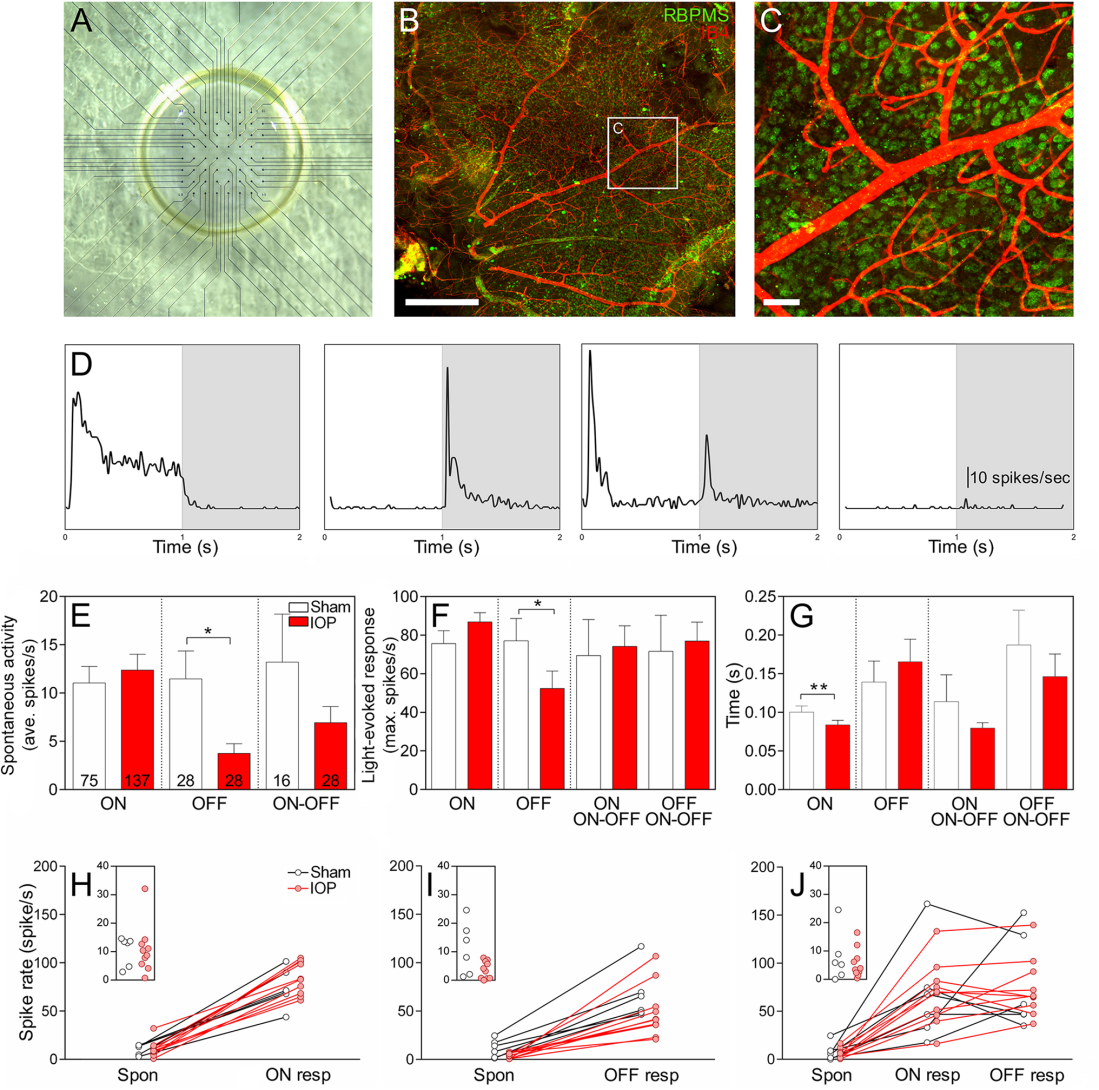
PMEA recordings of isolated WT mice retinae demonstrate a reduction in OFF-RGC responses and a reduction in ON-RGC latency following acute IOP elevation. The pMEA consists of a grid of electrodes, in which the retina rests RGC-side down with the aid of a vacuum pump that applies suction from below (**A**). Immunohistochemical staining after recording revealed little damage as the RGCs (RBPMS, green) and blood vessels (IB4, red) appeared intact ((**B**) scale bar 500 µm, magnified inset (**C**) scale bar 50 µm). (**D**) Representative peri-stimulus time histograms of RGC cell types in response to light on (white shading) or light off (grey shading). From the left to right of the panel, examples of an ON, OFF, ON–OFF and no-full-field-response cell are presented. (**E**–**G**) OFF-RGCs show a reduction in average spontaneous spike rate (**E**) and maximum spike rate (**F**) following elevated IOP (red bars) relative to sham eyes (clear bars). ON-RGCs show a reduction in latency to peak following elevated IOP (**G**). Numbers indicate the number of cells recorded from *n* = 6–10 animals. The spontaneous activity and light-elicited responses were averaged per retina and are presented for sham (black) and elevated IOP (red) for ON (**H**), OFF (**I**) and ON–OFF (**J**) RGCs. Insets show the spontaneous rate of cells from sham and elevated IOP eyes. *p < 0.05, **p < 0.01; Mann–Whitney test (sham vs IOP).

Inner retinal neuronal dysfunction has previously been shown to occur in acute IOP models^8,41,42^, however the effects of such an IOP injury on RGC types has not been evaluated. RGC responses were recorded using a pMEA (Fig. 2A), which induces minimal damage to the retina as the vasculature and RGC somata appear intact after recording (Fig. 2B,C). This methodology allowed us to assess the changes in many individual RGCs recorded simultaneously. Full field light was used to stimulate RGCs and classify them into cell types. ON- and OFF-RGCs were also divided into sustained and transient types and differences in the proportion of these cell classes investigated (Table S1). No effect of genotype or IOP treatment was observed on the proportions of sustained and transient cells recorded from, with the exception of ON–OFF RGCs, which showed a significant interaction but no individual differences between groups (Two way-ANOVA p = 0.03; Tukey’s multiple comparison’s post-test p > 0.05 for all; Table S1). Therefore sustained and transient types were combined for further analysis of spontaneous and light evoked responses in ON-, OFF- and ON–OFF RGCs. Representative PSTHs of RGC types in response to full field stimulation for ON-, OFF-, ON–OFF and no-full-field response (NFFR) are presented sequentially in the panel of Fig. 2D. Average data for individual cell responses from *n* = 6–10 animals, for spontaneous activity (Fig. 2E), maximal response to light stimuli (Fig. 2F) and time to maximal response (Fig. 2G) are presented for each RGC type. The spontaneous firing rate of OFF-RGCs was significantly reduced following elevated IOP compared to sham-treatment (3.76 ± 1.01 spikes/s IOP vs 11.47 ± 2.89 spikes/s sham, p = 0.035, *n* = 28 cells, Fig. 2E). In addition, the light-elicited firing rate of OFF-RGCs was reduced following acute IOP elevation compared to sham-treated OFF-RGCs (52.41 ± 9.06 spikes/s IOP vs 77.11 ± 11.48 spikes/s sham, p = 0.046, *n* = 28 cells, Fig. 2F). The spike rates of other cell classes were not affected, however there was a slight but significant decrease in the time to peak response in ON-RGCs following acute IOP elevation (0.084 ± 0.006 s IOP vs 0.100 ± 0.008 s sham, p = 0.001, *n* = 75–137 cells, Fig. 2G). To account for the possibility that a given animal may have contributed to the changes observed at the cell population level, spontaneous and light-elicited responses for each sham and IOP retinae were averaged for comparison and considered per eye (*n* = 6–10 animals). The spontaneous and maximum spike rate of OFF-RGCs was reduced in IOP eyes (red) relative to sham (black; Fig. 2I) whereas ON- (Fig. 2H) and ON–OFF (Fig. 2J) RGCs show similar responses between sham and IOP eyes.

**Figure 3:**
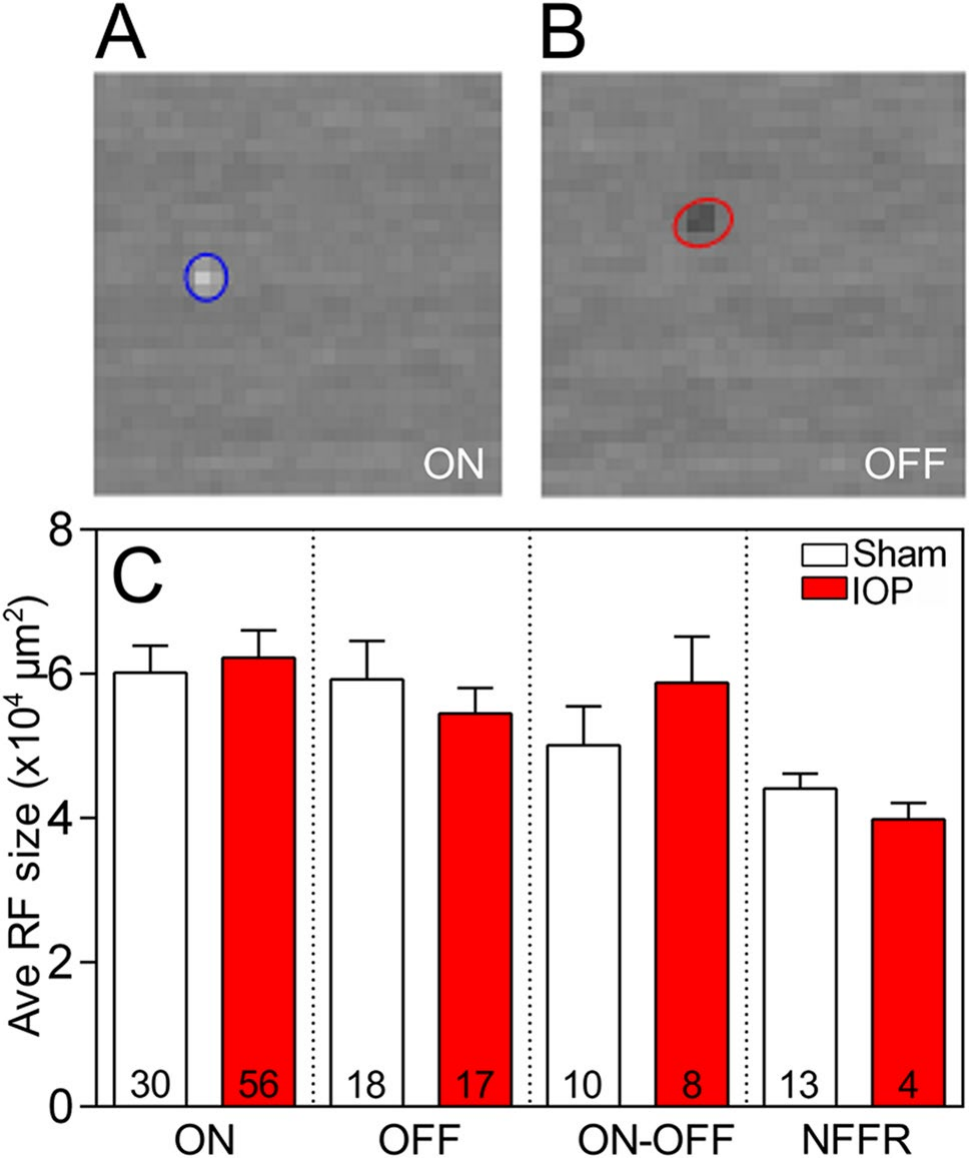
Receptive field size was not altered in any RGC type following acute IOP elevation. (**A, B**) The receptive field (RF) size calculated from the spike-triggered average of an ON (**A**) and OFF (**B**) RGC. Some cells that did not respond to the full field stimuli (NFFR cells) showed an STA response while others that were full field responsive had no STA, thus cells were still classified on their full field response and those with STAs compared. None of the RGC subtypes showed a change in receptive field size in sham (white bars) relative to IOP elevated (red bars) eyes (**C**). Numbers in histograms indicate number of cells recorded from *n* = 6–10 animals.

The receptive field sizes of RGCs were mapped using a 32 by 32 checkerboard stimulus from which the spike triggered average (STA) was calculated. Examples of an ON and OFF STA are presented in Fig. 3A,B, respectively. Some cells that did not respond to the full field stimuli (NFFR cells) showed an STA response, while some cells with a full-field response had no demonstrable STA response. For consistency with the previous analysis, cells were still classified by their full field response and only those cells with an STA were examined for changes in receptive field size. Receptive fields determined from STAs from NFFR cells were seemingly smaller than those of full field responsive cells, suggesting that these cells may have a small centre and strong antagonistic surround as has been noted in some OFF-RGCs in the mouse which have a strong response to spot but not full field stimuli^34^. STAs were not significantly different between RGC types between sham and treated eyes (Fig. 3C), suggesting acute IOP elevation had no effect on functional receptive field size measured using this technique three days after treatment (though we cannot rule out the possibility that changes smaller than 70 µm may have occurred).

### P2X7-R knockout does not rescue RGC function following acute IOP injury

**Figure 4:**
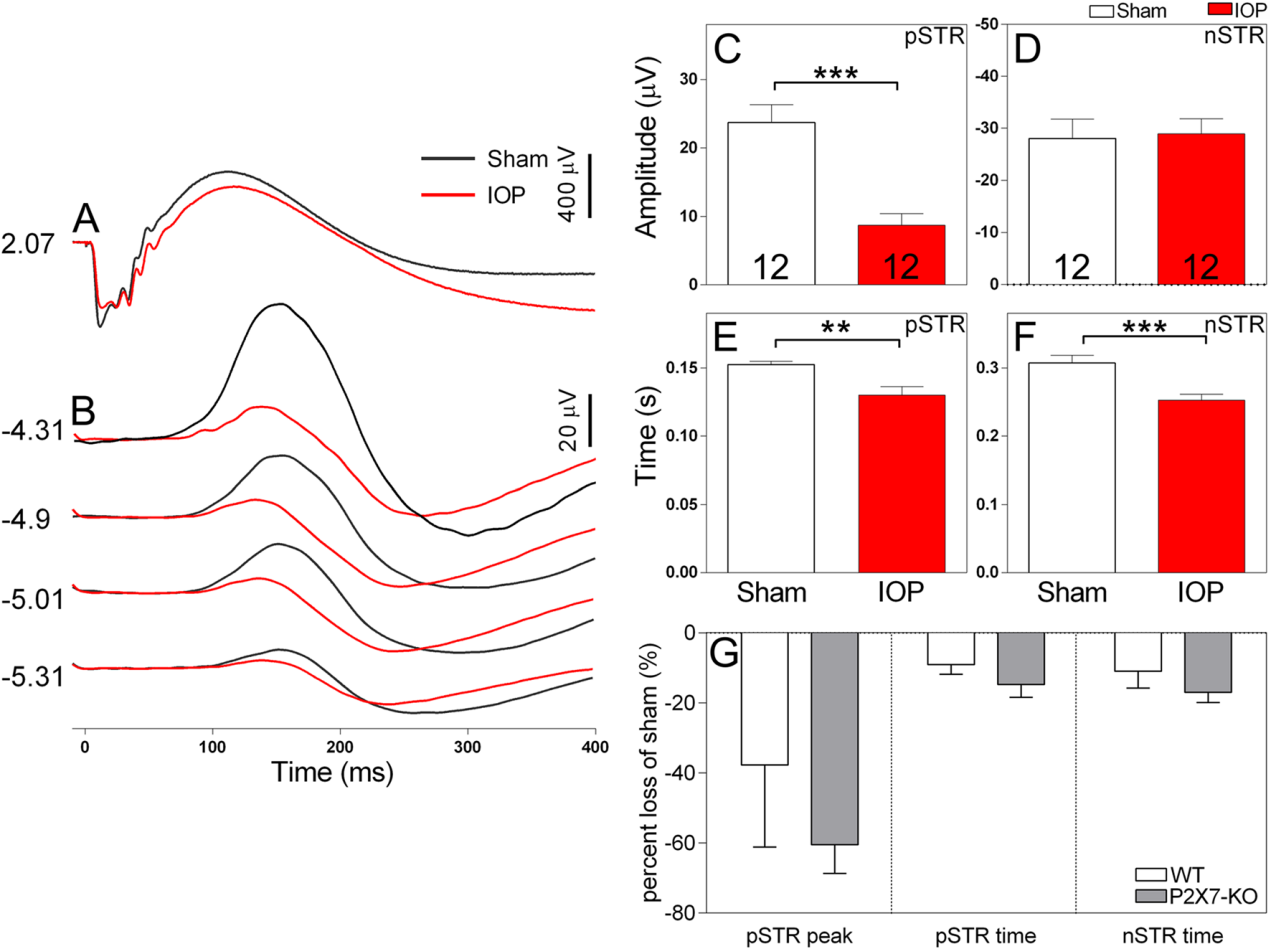
Electroretinogram recordings of P2X7-KO mice show a predominant reduction in the pSTR amplitude, without sign of rescue following acute IOP elevation. Representative waveforms of retinal responses measured at bright ((**A**), 2.07 log cd·s/m^2^) and dim ((**B**), − 5.31 to − 4.31 log cd·s/m^2^) luminous energies. Average amplitude of the pSTR (**C**) and nSTR (**D**) following sham (white bars) or elevated IOP (red bars) treatment. Average implicit time to the peak amplitude of the pSTR (**E**) or nSTR (**F**) following sham or elevated IOP treatment. The amplitude and implicit time of the pSTR was significantly reduced in eyes subject to elevated IOP. (**G**) The effect of IOP on pSTR and nSTR responses expressed as a percentage loss of the sham eye show no differences between P2X7-KO and WT. Numbers indicate the number of mice. **p < 0.01, ***p < 0.001; paired t-test (sham vs IOP).

The P2X7-R has been suggested to contribute to RGC death following increased IOP^9,43^. Therefore, we next wished to evaluate whether knock out of the P2X7-R contributes to recovery of function following elevated IOP. Our results demonstrate that genetic inactivation of P2X7-R does not ameliorate the effect of acute IOP elevation on summed, nor individual RGC responses. Full field ERG responses from sham and IOP-treated P2X7-KO animals are presented in Fig. 4. Like WT animals, the P2X7-KO mice showed reduced pSTR amplitude (8.73 ± 1.70 µV IOP vs 23.72 ± 2.61 µV sham, p = 0.0004, *n* = 12, Fig. 4C) and no change in nSTR amplitude (Fig. 4D) after elevated IOP. In addition, P2X7-KO animals showed reduced pSTR implicit time (0.130 ± 0.006 s IOP vs 0.152 ± 0.002 s sham, p = 0.001, *n* = 12, Fig. 4E) and nSTR implicit time (0.253 ± 0.009 s IOP vs 0.307 ± 0.011 s sham, p = 0.0003, *n* = 12, Fig. 4F) in eyes with elevated IOP. The effect of IOP on pSTR and nSTR responses were similar in WT and P2X7-KO eyes, with elevated IOP inducing a similar reduction in these responses in both animal strains (Fig. 4G). Further analysis of other waveform components of WT and P2X7-KO ERG responses following IOP elevation are summarized in Table 1. Of note, both genotypes displayed a significant reduction in a-wave sensitivity following IOP elevation and IOP treatment induced a significant reduction in b-wave amplitude in P2X7-KO animals. Overall, these results indicate that knockout of the P2X7-R does not prevent ERG dysfunction following increased IOP and the RGC response, as assessed by the pSTR, is not rescued.

**Figure 5:**
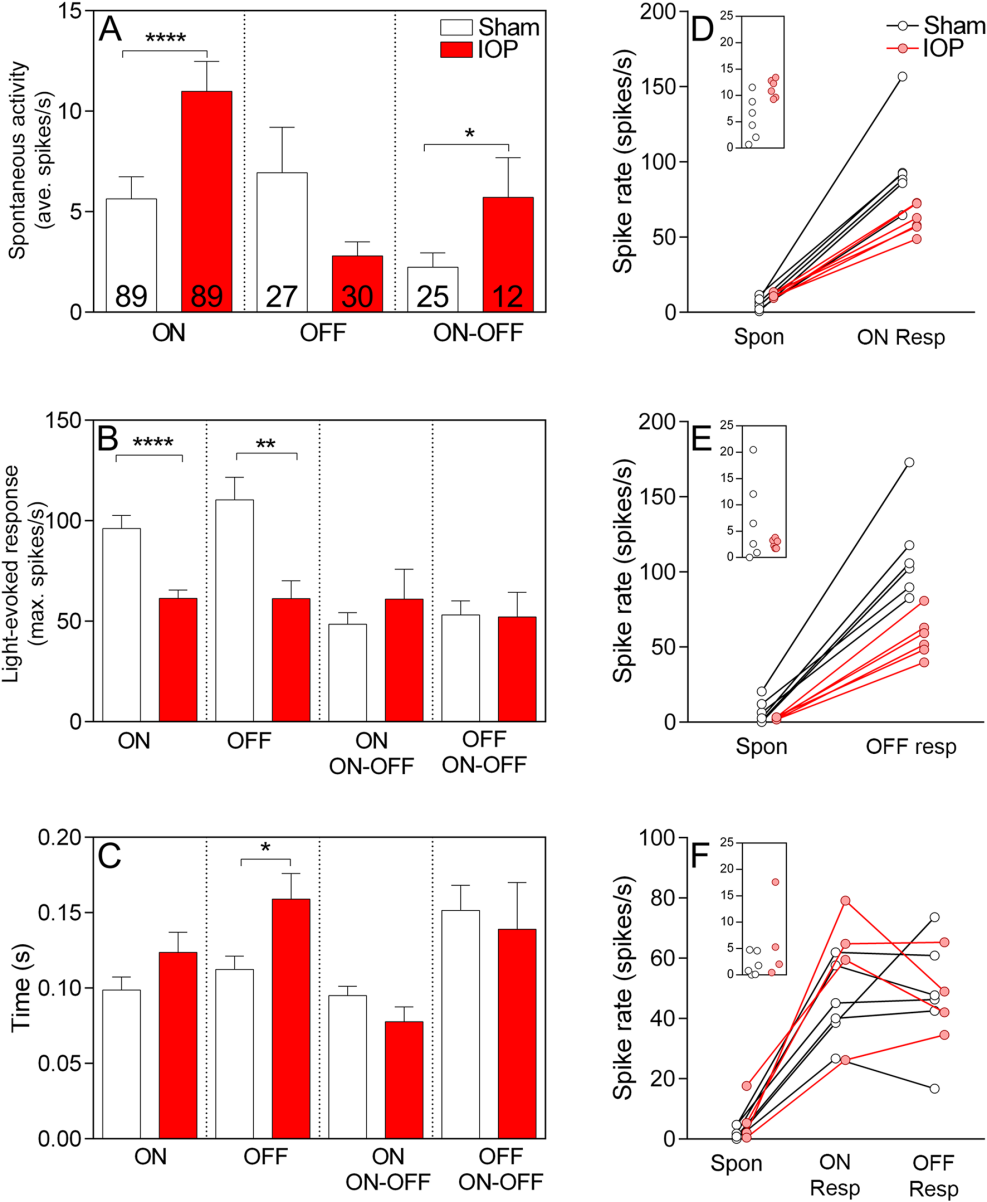
pMEA recordings of P2X7-KO mice demonstrate dysfunction of OFF-RGCs, without rescue, and additional dysfunction of ON-RGC following acute IOP elevation. (**A**) Spontaneous activity was increased in ON and ON–OFF RGCs and (**B**) light-elicited responses were reduced in ON- and OFF-RGCs following elevated IOP (red bars). (**C**) The latency to peak response was increased in OFF-RGCs following elevated IOP. Numbers indicate the number of cells recorded from *n* = 6 animals. (**D**–**F**) The spontaneous activity and light-elicited responses were averaged per retina and are presented per retina for ON- (**D**), OFF- (**E**) and ON–OFF (**F**) RGCs. Insets show the spontaneous rate of cells from sham and elevated IOP eyes. Both ON- and OFF-RGCs from the P2X7-KO mouse showed reduced light responsiveness following acute IOP elevation*p < 0.05, **p < 0.01, ****p < 0.0001; Mann–Whitney test (sham vs IOP).

The responses of individual RGCs from elevated IOP and sham retinae from the P2X7-KO mouse were assessed using the pMEA (Fig. 5). When examining the effect of IOP elevation, changes in the responses of ON-RGCs were also observed in the P2X7-KO in response to acute IOP elevation, unlike the WT retinae which primarily showed changes in OFF-RGCs. The average data for individual cell responses in *n* = 6 animals is shown (Fig. 5A–C).

The spontaneous activity of ON-P2X7-KO RGCs was significantly increased following elevated IOP compared to sham treatment (11.00 ± 1.48 spikes/s IOP vs 5.64 ± 1.10 spikes/s sham, p < 0.0001, *n* = 89 cells, Fig. 5A). Additionally, ON-RGC light responses were significantly reduced following IOP elevation (61.41 ± 4.22 spikes/s IOP vs 96.20 ± 6.46 spikes/s sham, p < 0.0001, *n* = 89 cells, Fig. 5B), with no change in latency of the response (Fig. 5C). These data suggest that the range of response of ON-RGCs is reduced by acute IOP elevation. For OFF-RGCs of P2X7-KO mice there was no significant effect on spontaneous activity (2.81 ± 0.70 spikes/s IOP vs 6.95 ± 2.27 spikes/s sham, p = 0.92, *n* = 27–30 cells, Fig. 5A). However, similarly to WT retinae, they displayed a reduced response to light following elevated IOP (61.26 ± 8.97 spikes/s IOP vs 110.4 ± 11.32 spikes/s sham, p = 0.002, *n* = 27–30 cells, Fig. 5B). The latency to reach peak spike rate was significantly increased in OFF-RGCs of P2X7-KO mice following IOP (0.16 ± 0.02 s IOP vs 0.11 ± 0.01 s sham, p = 0.02, *n* = 89, Fig. 5C). ON–OFF RGCs of P2X7-KO mice showed significantly increased spontaneous activity following elevated IOP (5.72 ± 1.96 IOP vs 2.24 ± 0.72 sham, p = 0.03, *n* = 12—25 cells, Fig. 5A), but showed no change in peak response rate with light stimulation (Fig. 5B) or time to peak (Fig. 5C). Spontaneous and light-elicited responses for each sham and IOP retinae were averaged for comparison and considered per eye (n = 6 animals; Fig. 5D–F). Figure 5D makes clear the reduced response range of ON-RGCs after IOP elevation. OFF-RGCs also show a reduced light-elicited response (Fig. 5E).

To ascertain if there were any baseline differences between WT and P2X7-KO responses, we compared the sham spontaneous activity, light-evoked responses and latency of ON, OFF and ON–OFF RGCs. Compared to WT, the P2X7-KO sham spontaneous responses were reduced in ON and ON–OFF RGCs (Table S3) and peak responses were increased in ON and OFF RGCs (Table S4). Latency to peak response was not different between WT and P2X7-KO sham-treated eyes (Table S5). These differences suggest the P2X7-KO RGCs have a greater response range compared to WT. Despite a greater range, IOP elevation still causes impaired RGC function in P2X7-KO retinae, with ON-RGCs showing the greatest effect. These findings suggest that the RGCs of P2X7-KO mice are not protected from acute IOP elevation and are instead more vulnerable to dysfunction, with ON-, OFF- and ON–OFF RGC responses affected.

### There are more P2X7-Rs in dendrites of ON cells than other RGC types in WT retinae

**Figure 6:**
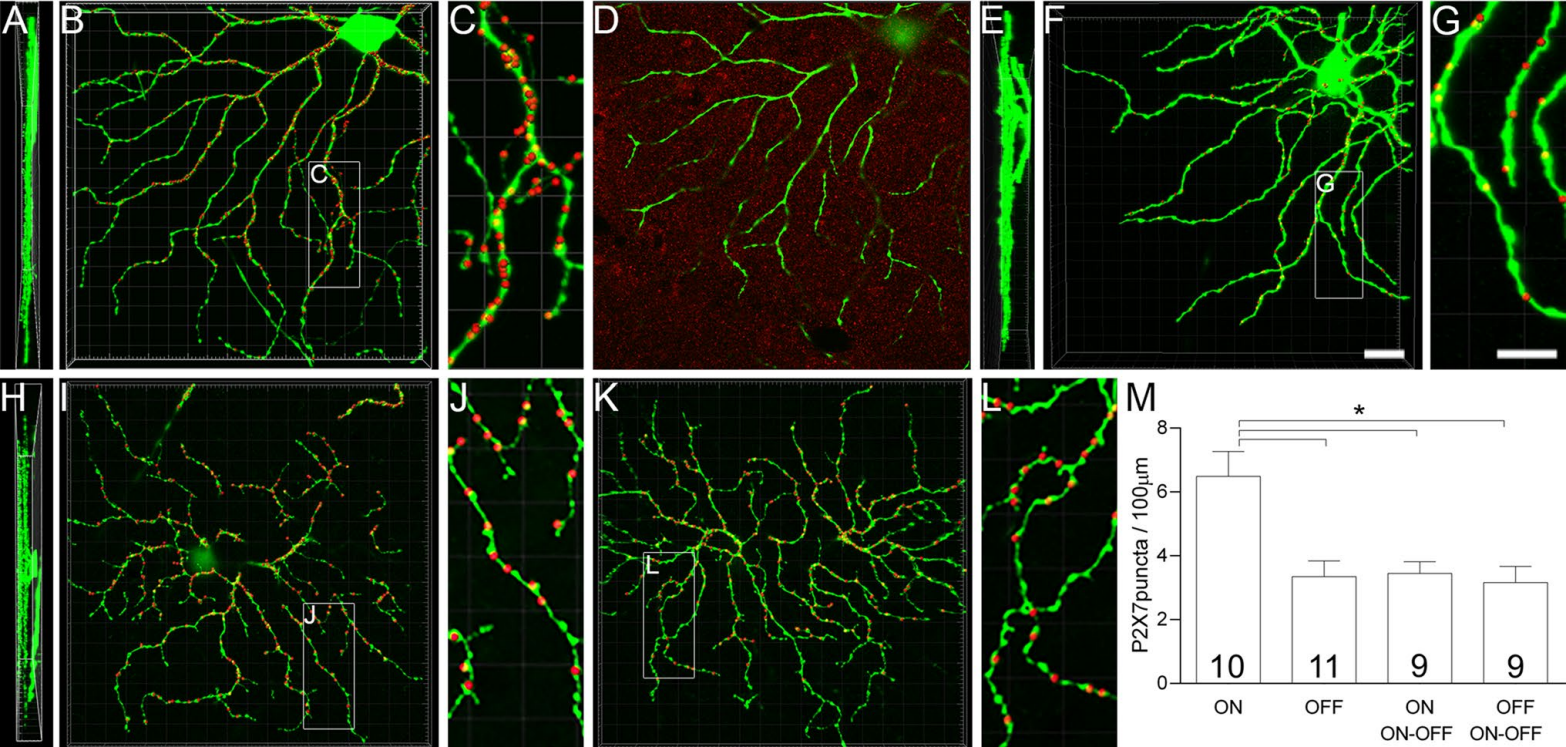
P2X7-Rs are highly expressed in ON-RGCs of Thy1-YFP-H mice. Thy1-YFP-H RGCs (green) are projections of the original confocal Z-stacks, while the P2X7-R puncta contacting the dendrites (red) are digitally rendered as spots (presented as 3 times larger than the true colocalised puncta for ease of viewing). (**A**–**C**) Thy1-YFP-H labelled ON-RGC (green) showing dense labelling for P2X7-R puncta (red) shown in (**A**) orthogonal view, (**B**) from above and (**C**) magnified inset from (**B**). (**D**) A single plane of raw data from the ON-cell (green) with P2X7-R staining (red). (**E**–**G**) Thy1-YFP-H labelled OFF-RGC (green) showing sparser labelling for P2X7-R puncta (red) than ON-RGCs. The OFF-RGC is shown in (**E**) orthogonal view, (**F**) from above and (**G**) magnified inset from **F**. (**H**–**L**) Thy1-YFP-H labelled ON–OFF RGC (green) in the orthogonal view (**H**) displaying dendritic fields stratifying in ON and OFF sublaminae. Labelling for the P2X7-R puncta (red) in the ON sublamina (**I**) from above and (**J**) magnified inset from (**I**). Labelling for the P2X7-R puncta (red) in the OFF sublamina (**K**) from above and (**L**) magnified inset from K. (**M**) Quantification of P2X7-R puncta in the ON and OFF lamina of the IPL for ON-, OFF- and ON–OFF RGCs. Numbers indicate number of cells from 4 animals. *p < 0.01; one-way ANOVA, Tukey’s multiple comparisons test. Scale bar is 10 µm.

One possible explanation for the vulnerability of ON-RGCs to IOP in the P2X7-KO is differential expression and localization of P2X7-Rs on these cells. We performed high resolution immunolocalization studies of the P2X7-R on RGC dendrites using Thy1-YFP-H mice. RGCs are only sparsely labelled in the Thy1-YFP-H mice allowing detailed analysis of individual ON-, OFF- and ON–OFF RGCs (Fig. 6). A one-way ANOVA revealed statistically significant differences between groups (F(3,35) = 8.005, p = 0.0003), and a Tukey’s post hoc analysis was performed. There were significantly more P2X7-R positive puncta localized to ON-RGC dendrites (6.49 ± 0.77 puncta/100 µm, Fig. 6M) compared to OFF- (3.35 ± 0.50 puncta/100 µm, p = 0.001, Fig. 6G) and ON–OFF RGCs in ON (3.45 ± 0.37 puncta/100 µm, p = 0.004) and OFF arbours (3.16 ± 0.50 puncta/100 µm, p = 0.001, Fig. 6M), which were examined separately. As dendritic arbours of ON- and ON–OFF RGCs are both found in the ON sublamina of the inner plexiform layer, the higher P2X7-R expression in ON-RGC dendrites is unlikely due to tissue processing or imaging confounds. The higher expression of the P2X7-R in ON-RGCs may explain why they show exacerbated dysfunction following injury in the P2X7-KO mice, indicating a role for the P2X7-R in maintaining ON-RGC function following acute IOP elevation.

### There is no loss of RGCs following IOP elevation

**Figure 7:**
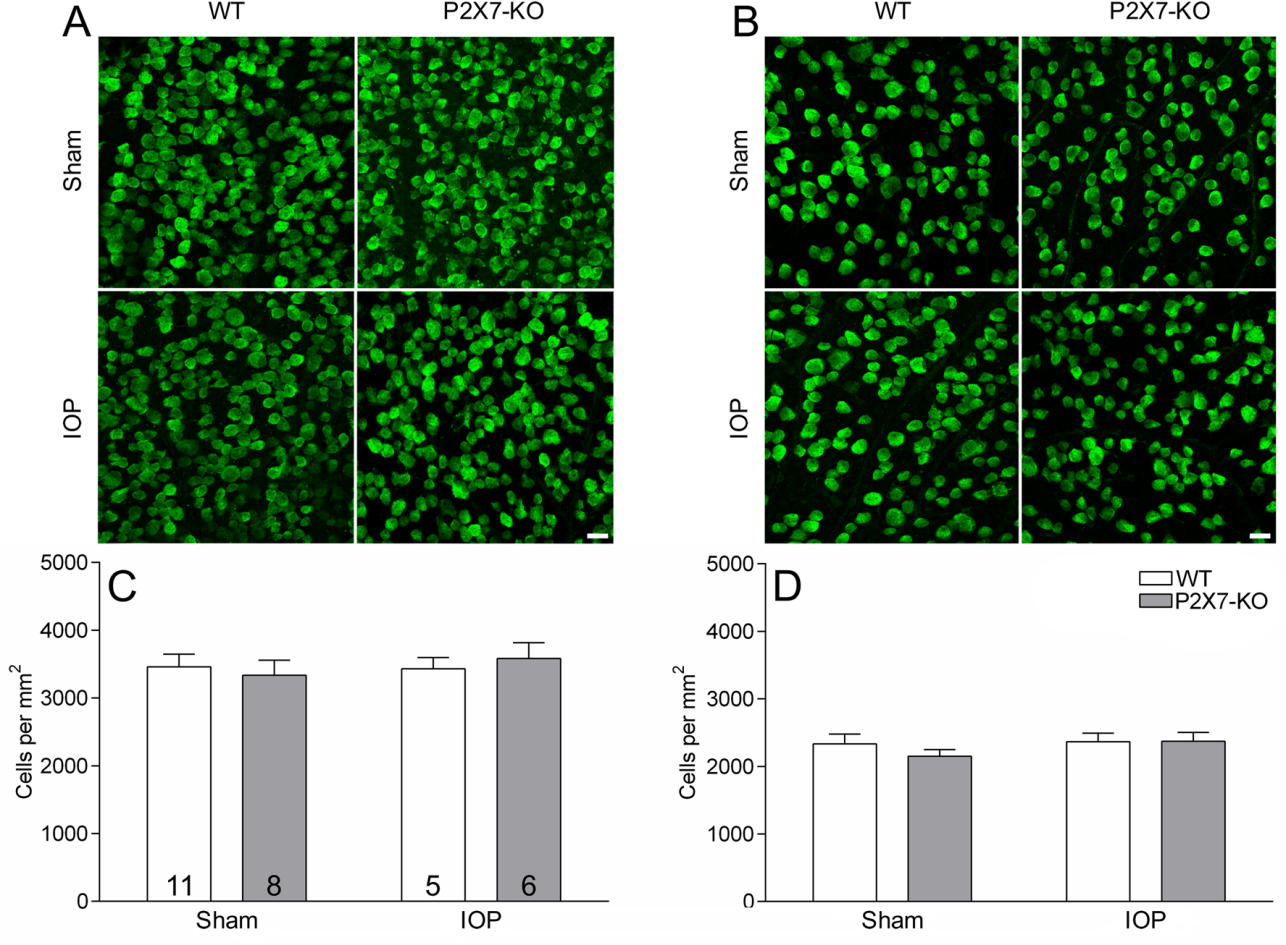
RGC numbers are not altered in either WT or P2X7-KO retinae following acute IOP elevation. RGCs were immunohistochemically labelled with RBPMS and images of representative staining of the central (**A**) and peripheral (**B**) eccentricities are shown. RGC counting showed no difference in RGC number between central (**C**) and peripheral (**D**) eccentricities. Numbers in histogram indicate number of animals. Scale bar 20 µm.

We have shown RGCs have reduced responsiveness to light 3 days following elevated IOP. This may be explained by RGC dysfunction or by the death of cells with high firing rates. Although cell death was considered to be unlikely given the use of a transient IOP model, retinae were stained with RBPMS (a pan-RGC marker) and cells were counted in central and peripheral eccentricities in sham and IOP retinae from WT and P2X7-KO mice to interrogate this possibility (Fig. 7). Images of RBPMS-labelled RGCs in central (Fig. 7A) and peripheral retina (Fig. 7B) from WT and P2X7-KO, sham and IOP eyes are presented showing similar labelling between IOP and sham treated eyes. Quantification of RGC numbers in WT and P2X7-KO mice indicated that there was no cell loss following increased IOP in central (Fig. 7C) or peripheral eccentricities (Fig. 7D) as compared to sham eyes. This confirms the acute IOP model has the potential to identify RGC dysfunction prior to death.

## Discussion

This study demonstrates that an acute increase in IOP is sufficient to cause RGC dysfunction. In WT mice, OFF-RGCs showed a reduction in activity 3 days after acute IOP stress, which occurred in the absence of cell loss. ON- and ON–OFF RGC light responses were minimally affected, and receptive field sizes for all RGC types were unaltered by IOP elevation. As the P2X7-R may play a role in RGC response to pressure, we hypothesised its removal may restore function following elevated IOP. In P2X7-KO mice, however, OFF-, ON–OFF, and particularly ON- RGCs showed dysfunctional light responsiveness after IOP elevation, suggesting the P2X7-R may serve an important role in maintaining ON-RGC function when the retina is subject to stress.

In the model of IOP elevation investigated here, the exact magnitude (50 mmHg) and duration (30 min) of IOP elevation was tightly controlled and non-ischemic. This allows us to investigate the response of RGCs to pressure over other, more severe models that rapidly induce RGC death^8^. Our ERG data indicate a prominent global loss of RGC function following IOP elevation, as measured by the pSTR, with minor changes to other retinal neuron responses. We also determined whether individual RGC types were specifically affected. OFF-RGC spontaneous activity and the light-elicited firing rate were reduced in WT mice after acute IOP elevation, while these responses in ON- and ON–OFF RGCs were not significantly altered. This supports previous studies that used more sustained IOP injury (3–30 days of elevated IOP) and demonstrated substantial OFF-RGC dysfunction, dendritic shrinkage and reduced receptive field size compared to other RGC types^44–46^. Further elucidation of how RGCs respond in rodent models following IOP injury is needed, however, as similar levels of dendritic shrinkage and altered RGC activity has been observed between ON-, OFF- and ON–OFF RGC types^47^.

Support for RGC type-specific losses exists in glaucoma patients as they show loss of larger RGCs and axons^48–50^. In addition, functional deficits of the larger magnocellular RGCs have been observed when probed with stimuli dissecting magnocellular and parvocellular function^51–54^, though the specificity with which such stimuli can isolate different pathways is disputed^55,56^.

In addition to the changes observed in OFF-RGCs in WT mice, a reduction in the latency of ON-RGC response to light was apparent following IOP elevation. In previous studies, RGC responses have been shown to have reduced latency following sustained and mild increases in IOP^57–59^. This may be due to damage to the AII amacrine cell, an inhibitory interneuron that can modulate the responses of ON- and OFF-RGCs, which exhibits disrupted activity following IOP, resulting in reduced latency of RGC firing^57,60^.

In contrast to the full field response, we saw no change in functional receptive field size of OFF-RGCs measured using the STA, which may be due to the acute IOP model or the post injury duration investigated here (3 days) and a structural change likely requires more time to take effect. This extends on previous studies where the earliest time point for examining dendritic field and receptive field changes was 7 days^44^.

The mechanisms underlying differences in type-specific RGC vulnerability to raised IOP is not well understood. It has been suggested that RGC types have differing receptor expression that could lead to varied responses to increased pressure^7^. The P2X7-R appears to show type-specificity as, in this study, we show greater immunocytochemical expression in ON-RGCs. A study examining P2X7-R function in ON and OFF pathways found greater contribution to the OFF pathway in the ERG response^61^. This was, however, studied in frog retinae and a difference in P2X7-R distribution between species may explain this conflicting finding. Indeed, Chavda et al.^62^ have found a greater effect of P2X7-R agonists and antagonists on the ON pathways of mice. Calcium-permeable glutamate receptors or associated proteins such as pannexin-1 and transient receptor potential vanilloid (TRPV) channels, have also been shown to be more highly expressed in distinct RGC types^63–65^. Activation of mechanosensitive channels, such as pannexin-1 and TRPV channels, with pressure or cellular swelling lead to calcium influx into the cell and may initiate RGC-type specific apoptosis and death^66–68^. Another potential mechanism leading to OFF-RGC type-specific vulnerability could relate to the structure of the retinal vasculature^46^. The dendrites of OFF-RGCs are in the distal layers of the inner plexiform layer, and are supplied by the intermediate capillary plexus and the intersublaminar vascular plexus^69^. Furthermore, OFF-RGCs have been shown to be functionally more excitable, suggesting they may be more metabolically demanding compared to ON cells and, thus, more sensitive to fluctuations in vascular supply^70,71^. These intrinsic and extrinsic factors likely combine to contribute to OFF-RGC vulnerability^7^.

We assessed whether the P2X7-KO could rescue the functional deficits caused by increased IOP due to evidence implicating this receptor during models of glaucomatous injury^72–75^. The knockout of P2X7-R revealed interesting, but unexpected findings, as ERG and MEA data confirmed the P2X7-KO did not prevent OFF-RGC dysfunction following acute IOP elevation. Although upregulation of P2X7-R gene and protein expression has been demonstrated in the DBA/2J genetic model of glaucoma^75^ and following an ischemic elevation of IOP^72^, these are more severe models compared to the one used in this study. In contrast, milder chronic elevation of IOP in WT mice has been shown to have no effect on P2X7-R gene expression^28^. In more severe IOP models resulting in cell death, an influx of inflammatory cells involved in clearing damage or inflammatory markers coincides with increased P2X7-R expression^72,73^. Reduction of neurotoxic inflammation by knockout or blockade of the P2X7-R may be one reason a beneficial effect against the damage caused by IOP was observed in these more severe models.

In the current study, P2X7-KO in conjunction with IOP injury led to added dysfunction of ON-RGCs compared to WT mice. The P2X7-R may be important for functional homeostasis of ON-RGCs, which is disrupted by acute IOP elevation. In the sham P2X7-KO retinae there were some differences from WT in spontaneous and peak response, which indicate that P2X7-KO RGCs appear to have a greater response range. ON and ON–OFF RGCs from P2X7-KO sham-treated eyes showed reduced spontaneous activity compared to sham WT eyes; and ON and OFF RGCs from P2X7-KO sham eyes showed increased peak light-evoked activity compared to sham WT eyes. Despite this, IOP elevation still caused a decline in RGC function in P2X7-KO retinae. Following elevated IOP, ON-RGCs displayed increased spontaneous activity and reduced light-elicited responses in P2X7-KO animals compared with IOP in WT animals, which can be interpreted as a loss of response range. ON–OFF RGCs of P2X7-KO mice showed an increase in spontaneous activity, without a corresponding increase in light-elicited response, which may also indicate a loss of response range. This may reduce contrast sensitivity (i.e. the gain) and impact the ability of ON-RGCs to adapt to diverse luminance levels. ON-sustained RGCs have displayed similar deficits in gain following a chronic model of IOP elevation^45^. Deficiencies in light adaptation have also been observed in glaucoma patients^76,77^.

In addition to RGCs, amacrine and bipolar cells may be vulnerable to elevated IOP as P2X7-KO animals demonstrate a reduction in the b-wave amplitude and nSTR latency. P2X7-Rs have been shown to play a functional role in maintaining the activity of amacrine cells that modulate the response of the b-wave^14^, thus its ablation may be detrimental to their function. As the P2X7-R is also found on microglia, amongst other retinal neurons^14^, the responses of these cells may also be affected in the P2X7-KO mouse. Microglial phagocytosis has been shown to be reduced in the retinae of P2X7-KO mice^78^ and blocking the P2X7-R has been shown to reduce retinal microglial activation during chronic IOP elevation^74^. Further work could elucidate whether impaired function of microglia may contribute to the greater level of dysfunction observed in P2X7-KO mice.

As ON-RGCs show higher P2X7-R expression it indicates the P2X7-R is important in maintaining normal function following acutely elevated IOP. There are also suggestions that the P2X7-R may have a neuroprotective role during death or injury in retinal and cerebellar neurons^79–82^. Another cell type to consider is the melanopsin-RGC (mRGC) which is more resistant to death following axotomy^83^ or acute, ischemic IOP injury^84^. The ON-RGCs may correspond to mRGCs as ON-sustained RGCs have been shown to stain weakly for melanopsin^85^. Therefore, it may be of interest to isolate the mRGC responses in the P2X7-KO to observe whether their responses are preserved. However, more research is required to understand these potentially protective mechanisms. Additional experiments using P2X7-R antagonists may also further validate the results observed in the present study. Despite the importance of understanding the mechanisms under sub-toxic conditions, further studies investigating the effect of P2X7-KO or antagonism in a more severe or chronic IOP injury would elucidate the role of the receptor following widespread cell death.

In conclusion, our study shows that OFF-RGCs show increased susceptibility to IOP elevation, and the effects of even a transient, acute increase can be observed 3 days after increased IOP. Despite RGC dysfunction, the cells are not lost at this early stage. Our study also suggests that the P2X7-R may play an important role in ON-RGCs as the ablation of the P2X7-R reduces their ability to respond to acute pressure injury. Our data suggest the role of P2X7-Rs during the initial stages of IOP injury may differ from its role during sustained or ischemic IOP injury, and suggests treatments targeting this receptor may need careful consideration. Further investigations of mechanisms contributing to RGC-specific vulnerability will be important in understanding glaucoma progression and will inform the development of future treatments.

## Supplementary Information

Supplementary Information.
