# Supplementary material for: Retinal ganglion cell dysfunction in mice following acute intraocular pressure is exacerbated by P2X7 receptor knockout
Source: Sci Rep. 2021 Feb 18;11:4184. doi: 10.1038/s41598-021-83669-0 (PMC7893065; doi:10.1038/s41598-021-83669-0)
Supplement: Supplementary file 1 — Supplementary Information. [file 41598_2021_83669_MOESM1_ESM.docx]

**Supplementary material**

**Title:** Retinal ganglion cell dysfunction in mice following acute intraocular pressure is exacerbated by P2X7 receptor knockout

**Authors:** Anna Y. M. Wang^1^, Vickie H. Y. Wong^2^, Pei Ying Lee^2^, Bang V. Bui^2^, Stefanie Dudczig^1^, Kirstan A. Vessey^1^*, Erica L. Fletcher^1^*

*Co-last authors

**Author affiliations:**

^1^Department of Anatomy & Neuroscience, University of Melbourne, Australia, 3010

^2^Department of Optometry and Vision Sciences, University of Melbourne, Australia, 3010

**Corresponding author email address:** [e.fletcher@unimelb.edu.au](mailto:e.fletcher@unimelb.edu.au)

**Number of supplementary pages (not including title page):** 2

**Number of supplementary tables:** 5

Supplementary Table S1. The proportion of RGC types show no significant difference amongst WT and P2X7-KO sham and IOP-treated groups

| RGC type | **Proportion of cells (%)** | | | |
| --- | --- | --- | --- | --- |
|  | **WT** | | **P2X7-KO** | |
|  | **Sham** | **IOP** | **Sham** | **IOP** |
| ON-transient | 32.00 + 6.03 | 38.20 + 3.57 | 37.28 + 8.23 | 42.81 + 5.66 |
| ON-sustained | 17.12 + 5.04 | 15.36 + 5.18 | 18.29 + 6.11 | 12.99 + 4.85 |
| OFF-transient | 12.00 + 3.72 | 10.04 + 2.30 | 13.61 + 1.59 | 16.62 + 2.36 |
| OFF-sustained | 5.23 + 3.72 | 1.88 + 0.70 | 2.12 + 0.95 | 1.65 + 1.07 |
| ON-OFF | 10.19 + 3.02 | 13.19 + 2.49 | 15.95 + 2.73 | 6.67 + 2.70 |
| NFFR | 23.47 + 5.58 | 21.34 + 3.98 | 12.75 + 3.14 | 19.27 + 3.34 |

Supplementary Table S2. ERG measures of naïve and Sham WT retinae were compared with no significant differences.

| ERG component | Naïve (n = 6) | Sham (n = 12) | *p-value* |
| --- | --- | --- | --- |
| A-wave amplitude | -527.4 + 56.86 | -519.4 + 29.70 | 0.89 |
| A-wave sensitivity | 3.05 ± 0.03 | 3.03 ± 0.05 | 0.69 |
| B-wave amplitude | 661.1 ± 66.69 | 692.2 ± 37.49 | 0.67 |
| B-wave sensitivity | -2.38 ± 0.05 | -2.39 ± 0.08 | 0.67 |
| pSTR amplitude | 15.11 ± 1.78 | 16.94 ± 2.13 | 0.59 |
| pSTR implicit time | 160.0 ± 4.64 | 155.1 ± 4.24 | 0.48 |
| nSTR amplitude | -16.57 ± 1.96 | -22.44 ± 3.03 | 0.22 |
| nSTR implicit time | 300.8 ± 14.01 | 292.0 ± 9.39 | 0.60 |

Supplementary Table S3. MEA measures of spontaneous activity (average spikes/sec) in sham-treated WT and P2X7-KO retinae. **p = 0.0072, ***p = 0.0005 compared to WT sham.

|  | **WT Sham** | **P2X7-KO Sham** |
| --- | --- | --- |
| ON | 11.04 + 1.72 | 5.64 + 1.10** |
| OFF | 11.47 *+* 2.89 | 6.95 + 2.27 |
| ON-OFF | 13.21 + 4.97 | 2.24 + 0.72*** |

Supplementary Table S4. MEA measures of peak light-elicited activity (maximum spikes/sec) in sham-treated WT and P2X7-KO retinae. **p = 0.0075, *p = 0.0263 compared to WT sham.

|  | **WT Sham** | **P2X7-KO Sham** |
| --- | --- | --- |
| ON | 75.65 + 6.77 | 96.20 + 6.462** |
| OFF | 77.11 + 11.48 | 110.4 + 11.32* |
| ON-OFF (ON) | 69.43 + 18.66 | 48.62 + 5.17 |
| ON-OFF (OFF) | 71.64 + 18.71 | 53.25 + 6.97 |

Supplementary Table S5. MEA measures of latency (ms) to peak light-elicited activity in sham-treated WT and P2X7-KO retinae.

|  | **WT Sham** | **P2X7-KO Sham** |
| --- | --- | --- |
| ON | 100.2 + 8.239 | 98.75 + 8.616 |
| OFF | 139.2 + 27.25 | 112.4 + 8.824 |
| ON-OFF (ON) | 114.1 + 34.54 | 95.15 + 6.041 |
| ON-OFF (OFF) | 187.4 + 44.69 | 151.6 + 16.56 |
